# New cell culture model for aromatase inhibitor-resistant breast cancer shows sensitivity to fulvestrant treatment and cross-resistance between letrozole and exemestane

**DOI:** 10.3892/ijo.2015.2850

**Published:** 2015-01-26

**Authors:** STINE HOLE, ASTRID M. PEDERSEN, SUSANNE K. HANSEN, JOHAN LUNDQVIST, CHRISTINA W. YDE, ANNE E. LYKKESFELDT

**Affiliations:** 1 Breast Cancer Group, Cell Death and Metabolism, Danish Cancer Society Research Center, DK-2100 Copenhagen Ø, Denmark; 2 Department of Biomedicine and Veterinary Public Health, Swedish University of Agricultural Sciences, Box 7028, SE-750 07 Uppsala, Sweden

**Keywords:** breast cancer model, aromatase inhibitor resistance, estrogen receptor, treatment options, fulvestrant

## Abstract

Aromatase inhibitor (AI) treatment is first-line systemic treatment for the majority of postmenopausal breast cancer patients with estrogen receptor (ER)-positive primary tumor. Although many patients benefit from treatment, some will develop resistance, and models mimicking acquired resistance will be valuable tools to unravel the resistance mechanisms and to find new treatments and biomarkers. Cell culture models for acquired resistance to the three clinically relevant AIs letrozole, anastrozole and exemestane were developed by selection and expansion of colonies of MCF-7 breast cancer cells surviving long-term AI treatment under conditions where endogenous aromatase-mediated conversion of androgen to estrogen was required for growth. Four cell lines resistant to each of the AIs were established and characterized. Maintenance of ER expression and function was a general finding, but ER loss was seen in one of twelve cell lines. HER receptor expression was increased, in particular EGFR expression in letrozole-resistant cell lines. The AI-resistant cell lines had acquired ability to grow without aromatase-mediated conversion of testosterone to estradiol, but upon withdrawal of AI treatment, testosterone induced minor growth stimulation. Letrozole, exemestane and tamoxifen were able to abrogate the testosterone stimulation but could not reduce growth to below the level in standard growth medium with AI, demonstrating cross-resistance between letrozole, exemestane and tamoxifen. In contrast, fulvestrant totally blocked growth of the AI resistant cell lines both after withdrawal of AI and with AI treatment. These data show that ER is the main driver of growth of the AI-resistant cell lines and indicate ligand-independent activation of ER. Fulvestrant is an efficient treatment option for these AI-resistant breast cancer cells, and the cell lines will be useful tools to disclose the underlying molecular mechanism for resistance to the different AIs.

## Introduction

Endocrine therapy is up-front systemic therapy for breast cancer patients with estrogen receptor (ER)-positive tumors. The antiestrogen tamoxifen has been the key endocrine agent since the 1970s and the meta-analysis after 15 years follow-up of 5 years adjuvant tamoxifen therapy revealed reduction in both recurrence risk and death (1). The third-generation aromatase inhibitors (AIs) letrozole, anastrozole and exemestane were introduced in the 1990s and proved superior to tamoxifen both in advanced disease and in the adjuvant setting (2–5). Consequently, the recommended first-line endocrine therapy for postmenopausal women with ER-positive disease is treatment with a third-generation AI (6). Both primary (*de novo*) and secondary (acquired) resistance occur, limiting the benefit of AI therapy. This emphasizes the need for early identification of resistance and new treatment options for patients with resistant tumors. Clinical data demonstrating improved outcome by combining AI therapy with HER2 targeted therapy or with PI3K/AKT/mTOR inhibitors (7–9) show that these pathways play important roles in AI-resistant cell growth. However, at present there are no tools to select the patients for specific therapy, and the heterogeneity of clinical resistance (10,11) underscores the need for extensive clinical and basic research to disclose the underlying molecular mechanisms for resistance to AIs.

The estrogen supply for postmenopausal breast tumors originates from circulatory uptake and local synthesis in both tumor cells and surrounding tissue, e.g., adipocytes, fibroblasts and inflammatory cells (12–15). The resistance mechanisms to AIs may differ in tumors depending on systemic estrogen supply and tumors in which the carcinoma cells are able to utilize the endogenous aromatase enzyme for estrogen supply. Cell models, mimicking AI resistance in tumors depending on systemically delivered estrogen, have been established from estrogen-responsive breast cancer cells, which have been adapted to grow under long-term estrogen deprivation (LTED) (16–20). LTED cells grow estrogen independently and cell growth has been shown to occur primarily via cross-talk between ER and growth factor receptor signaling pathways, including HER2, IGF-IR and PI3K (21–26). AIs have no effect on growth of LTED cells, whereas antiestrogens, and in particular the ER down modulator fulvestrant, inhibit growth of LTED cells (21,22,27). The importance of local synthesis versus circulatory uptake for supply of estrogen to the cancer cells has been debated and although most breast carcinoma cells express aromatase, most studies point towards a major importance of uptake of circulatory estrogen (13–15). Treatment with AIs results in total suppression of whole body estrogen synthesis, but the cancer cells have the capability to increase local estrogen production. Therefore, a model with endogenous estrogen synthesis is warranted.

Surrogate models with aromatase overexpressing breast cancer cells have been developed by stable introduction of aromatase cDNA (25,28–30). Cell lines and xenografts with acquired resistance to AIs have been established by long-term AI treatment of MCF-7 and T47D cells with exogenous over-expression of aromatase (25,31,32). Genome-wide analyses have revealed that the expression profiles for the MCF-7 sublines resistant to the non-steroidal compounds letrozole and anastrozole were very similar whereas a different profile was observed in cell lines with acquired resistance to the steroidal inhibitor exemestane (31,33). Ligand-independent activation of ER was found in both letrozole and anastrozole-resistant cell lines (31), whereas exemestane appeared to act as a weak agonist in exemestane-resistant cell lines, resulting in e.g., induction of the EGFR ligand amphiregulin and activation of EGFR signaling (33,34). A xenograft model with aromatase overexpressing breast cancer cells has disclosed adaptive changes resulting in activation of alternate signaling pathways due to increased expression of e.g., EGFR, HER2 and IGF-IR (25).

The regulation of the endogenous aromatase gene (*CYP19A1*) is very complex (12) and may play a key role in AI resistance. We have discovered culture conditions for MCF-7 cells, under which the growth is dependent on conversion of androgen to estrogen via the endogenous aromatase enzyme (35). Treatment with AIs totally suppressed cell growth under these culture conditions (35,36). However, during long-term treatment with AIs, a small subpopulation of the cells survived and slowly resumed growth. From such cells, we have been able to establish AI-resistant cell lines. These cell lines are unique models, which mimic acquired AI resistance in tumors utilizing endogenous aromatase activity to obtain estrogen stimulated cell growth. This report is an initial characterization of our panel of letrozole-, exemestane- and anastrozole-resistant cell lines, presenting the expression of HER receptors, ER and ER-regulated proteins, and growth response to treatment with AIs, tamoxifen and fulvestrant.

## Materials and methods

### Cell lines and culture conditions

The parental cell line for the AI-resistant cell lines was MCF-7 subline 0.5 (MCF-7/S0.5), which originates from MCF-7 cells from the Human Cell Culture Bank (Mason Research Institute, Rockville, MD, USA) that have been stepwise adapted to grow with 0.5% fetal calf serum (FCS) (37). The MCF-7/S0.5 (MCF-7) cells were maintained at 37°C in humidified air with 5% CO_2_ in phenol red-free DMEM/F12 medium (Life Technologies, Carlsbad, CA, USA) supplemented with 1% heat inactivated FCS (Life Technologies), 2 mM GlutaMAX™-1 (Life Technologies) and 6 ng/ml insulin (Sigma-Aldrich, St. Louis, MO, USA). In order to obtain growth, which depends on conversion of androgen to estrogen via the endogenous aromatase enzyme, MCF-7 cells were transferred to medium with 10% newborn calf serum (NCS) (Life Technologies) and 10^−7^ M testosterone (Sigma-Aldrich) as described previously (35). AI-resistant cell lines were established from MCF-7 cells grown in medium with 10% NCS and 10^−7^ M testosterone. A culture of MCF-7 cells were treated with AI [10^−6^ M letrozole, 10^−7^ M anastrozole or 10^−7^ M exemetane (Selleck Chemicals, Munich, Germany)] for one week, trypsinized and seeded in serial dilutions in 24-well plates. Single colonies were transferred to new wells and gradually expanded in medium with AI. After ~2–3 months, the isolated colonies gave rise to AI-resistant cell lines, which could be grown in AI-containing medium with a weekly split ratio of ~1:25. The MCF-7 cell line was authenticated in January 2014 by DNA profiling using short tandem repeat loci performed by Leibniz-Institut DSMZ (Braunschweig, Germany) and found to be matching the genetic profile reported for the MCF-7 cell line (DSMZ ACC 115).

### Growth experiments

For dose-response growth experiments, MCF-7 cells were seeded in 24-well plates (Nunc) in their standard growth medium and after one day, they were switched from 1% FCS to 10% NCS. On day 2, treatment was initiated and renewed on day 5. On day 7, cell number was determined by a crystal violet colorimetric assay (38). One week prior to the dose-response growth experiments, AI and testosterone were withdrawn from the growth medium for resistant cell lines. The AI-resistant cell lines were seeded in medium with 10% NCS, experimental medium added on day 2, renewed on day 5 and cell number determined on day 7 as described above. For combination of AI and antiestrogen treatment, the AI-resistant cell lines were seeded in their standard growth medium and treatment from days 2 to 7 with 10^−6^ M tamoxifen (Sigma-Aldrich) and 10^−7^ M fulvestrant (ICI 182.780; Tocris Bioscience, Bristol, UK) was performed in standard growth medium with the respective AI. All growth experiments were performed with four sample replicates or more and repeated at least twice with similar results.

### Western blot analysis

Lysates (RIPA buffer; 100 nM NaCl, 20 mM Tris base, 1% Triton X-100, 0.5% sodium deoxycholate, 0.1% SDS, 1 nM EDTA, pH 8.0) were prepared from early passages (5 and 6) of four cell lines resistant to each of the three AIs; letrozole, anastrozole and exemestane grown in their standard growth medium and from the selected AI-resistant cell lines; Let^R^-1, Let^R^-3, Exe^R^-1 and Exe^R^-3 grown in standard growth medium in passages between 15–30. Lysates from MCF-7 cells grown in standard growth medium with 1% FCS or grown for 21 weeks with 10% NCS + 10^−7^ M testosterone were used for comparison. For analysis of estrogen-regulated proteins, lysates were prepared from MCF-7 cells and from AI-resistant cell lines (withdrawn from AI and testosterone for one week) grown for five days with 10% NCS and 10% NCS supplemented with estradiol (Sigma-Aldrich), testosterone and testosterone in combination with AIs. SDS-PAGE page and immunoblotting were performed as described previously (39). Antibodies used against the proteins were: β-actin (A5441) from Sigma-Aldrich); Bcl-2 (M0887), EGFR (M7298), HER2 (A0485) and HER3 (M7297) from Dako (Glostrup, Denmark); ERα (RM-9101), progesterone receptor (PR-A and PR-B) (RM-9102) and Hsp70 (MS-482-PO) from Neomarkers (Fremont, CA, USA) and HER4 (4795) from Cell Signaling Technology (Danvers, MA, USA). Blots were washed four times with TBS/0.1% Tween-20 followed by incubation for 1 h with species-specific peroxidase-conjugated secondary antibodies (Dako). Detection was done using ECLplus reagent (GE Healthcare) and a Fujifilm image reader (LAS1000).

### Quantitative RT-PCR

MCF-7 cells were grown with 10% NCS + 10^−7^ M testosterone for five days and AI-resistant cell lines were grown in their standard medium. RNA was isolated using PureLink Micro-to-Midi Total RNA Purification system (Life Technologies) and reverse transcribed to cDNA by the High-Capacity RNA-to-cDNA kit (Life Technologies). The real-time PCR analysis was performed with Power SYBR^®^ Green PCR Master Mix (Life Technologies) using a Rotor-Gene 3000 (Corbett Life Science, Sidney, Australia). All experiments were conducted in accordance to the manufacturer’s recommendations. Results were calculated based on a real-time RT-PCR relative quantification strategy and presented as mean relative gene expression levels, compared to the parental MCF-7 cells. Primers and programs for CYP19A1 were as previously described by Díaz-Cruz *et al* (40).

### Statistical analysis

Two-tailed t-test with Bonferroni adjusted p-values for multiple group comparisons was used. The level of statistical significance was set to p<0.05, and indicated by asterisks in the figures.

## Results

### Testosterone stimulation of MCF-7 cells

**Figure 1:**
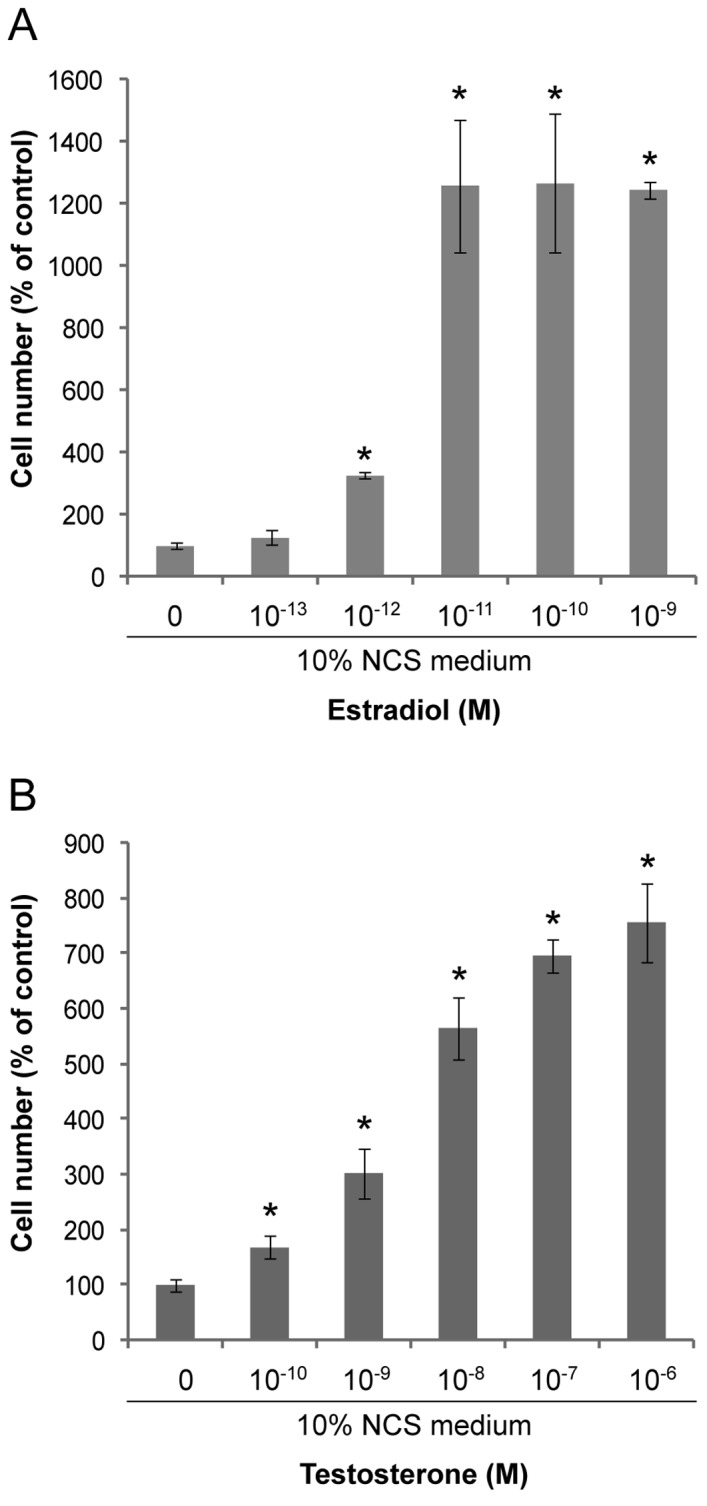
Effect of estradiol and testosterone on growth of MCF-7 cells. MCF-7 cells were cultured for five days in medium with 10% NCS and the indicated concentrations of estradiol (A) or testosterone (B). Cell number was estimated by a colorimetric assay and expressed relative to the NCS control culture. Results from one of two independent experiments with four sample replicates are shown. Mean and SD are shown and the asterisks indicate statistically significant difference from the NCS culture.

To study the effect of AIs and acquired AI resistance, a model system in which cell growth is stimulated by estradiol produced via aromatase-mediated conversion of testosterone is required. Newborn calf serum (NCS) contains low amount of estrogenic activity and MCF-7 cells require estrogen supplementation to grow continuously in 10% NCS (35). Both estradiol and testosterone exerted dose-dependent growth stimulation of MCF-7 cells in medium with 10% NCS (Fig. 1). Maximal growth stimulation of 13-fold was obtained with estradiol concentrations from 10^−11^ M (Fig. 1A), whereas maximal stimulation of 8-fold was seen with testosterone in concentrations of 0.1–1.0 μM (Fig. 1B).

### Establishment of AI-resistant cell lines and determination of ER, PR, Bcl-2, HER receptors and CYP19A1 mRNA

**Figure 2:**
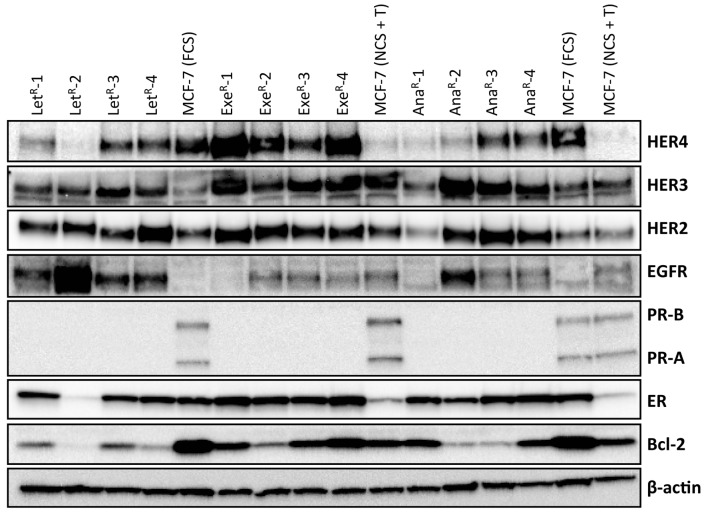
Expression of ER, PR, Bcl-2 and HER receptors in MCF-7 and AI-resistant cell lines. Western blot analysis of protein expression in AI-resistant cell lines grown under standard conditions with their respective AI and MCF-7 cells grown with 1% FCS or grown with 10% NCS + 10^−7^ M testosterone for 21 weeks. β-actin was used as loading control.

**Figure 3:**
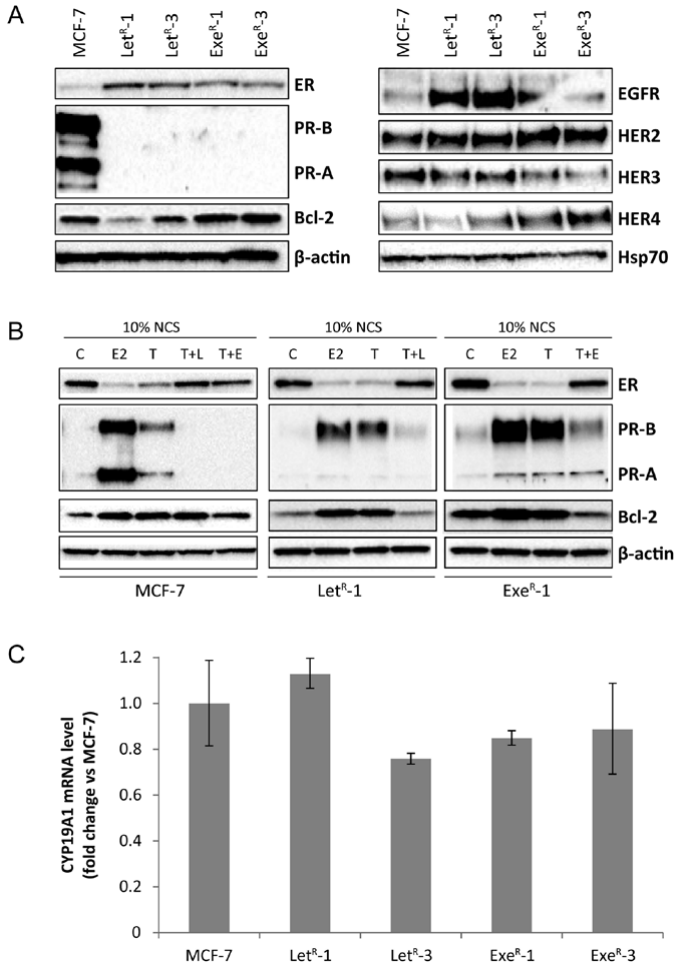
Expression of ER, PR, Bcl-2, HER receptors and CYP19A1 mRNA in MCF-7 and AI-resistant cell lines. (A) Western blot analysis of lysates from MCF-7 cells grown with 10% NCS + 10^−7^ M testosterone (MCF-7) and Let^R^-1, Let^R^-3, Exe^R^-1 and Exe^R^-3 grown in their standard growth medium with 10^−6^ M letrozole and 10^−7^ M exemestane, respectively. β-actin and Hsp70 were used as loading controls. (B) Western blot analysis of lysates from MCF-7, Let^R^-1 and Exe^R^-1 cells grown for five days in 10% NCS (C) or 10% NCS + 10^−12^ M estradiol (E2), 10% NCS + 10^−7^ M testosterone (T), 10% NCS + 10^−7^ M testosterone + 10^−6^ M letrozole (T + L), 10% NCS + 10^−7^ M testosterone + 10^−7^ M exemestane (T+E). Let^R^-1 and Exe^R^-1 cells were withdrawn from testosterone and their respective AI one week before onset of experiment. β-actin was used as loading control. (C) CYP19A1 mRNA level in MCF-7 cells grown with 10% NCS + 10^−7^ M testosterone for five days and AI-resistant cell lines grown in their standard medium determined by quantitative RT-PCR.

The testosterone stimulation of MCF-7 cell growth can be completely abrogated by addition of the third-generation AIs, letrozole, anastrozole and exemestane (36), but after long-term treatment colonies of cells grow out. We have selected four cell lines resistant to each of the three AIs, letrozole, anastrozole and exemestane, from isolated single colonies from cultures treated for long-term (≥2 months) with 10^−6^ M letrozole, 10^−7^ M anastrozole and 10^−7^ M exemestane, respectively (see Materials and methods). An initial analysis for expression of ER and the ER-regulated proteins; progesterone receptor (PR-A and PR-B) and Bcl-2 as well as the HER receptors, was performed on the cells harvested after 2.5 months with the respective AI (Fig. 2). All but one AI-resistant cell line maintained ER expression and the level of ER was comparable or higher than in parental MCF-7 grown with 1% FCS. MCF-7 cells grown with 10% NCS + 10^−7^ M testosterone had very low level of ER (Fig. 3B). PR-B and PR-A were not detectable in the resistant cell lines which were grown continuously in medium with testosterone and AI (Fig. 2). Bcl-2 level was lower in resistant cell lines than in MCF-7 cells grown under standard conditions with 1% FCS. EGFR level was low in MCF-7 cells and also in exemestane-resistant cell lines, whereas increased level of EGFR was seen in all four letrozole-resistant cell lines and in one anastrozole-resistant cell line. Noteworthy, the letrozole-resistant cell line with highest EGFR expression had a very low ER level. HER2 and HER3 were increased in most AI-resistant cell lines compared with MCF-7. In contrast, HER4 was reduced in letrozole- and anastrozole-resistant cell lines compared with parental MCF-7 cells grown in 1% FCS, whereas HER4 was increased in exemestane-resistant cell lines.

For further analyses, we selected two cell lines resistant to the non-steroidal AI letrozole, Let^R^-1 and Let^R^-3, and two cell lines resistant to the steroidal AI exemestane, Exe^R^-1 and Exe^R^-3. Initially, we tested the stability regarding expression of ER, PR, Bcl-2 and HER receptors. Fig. 3A is a representative experiment with determination of protein expression in AI-resistant cells from passages in which they have obtained stable growth rates in presence of the respective AI. The resistant cell lines maintained ER expression and the level of ER was higher in both the letrozole- and exemestane-resistant cell lines compared with MCF-7 cells grown with 10% NCS + 10^−7^ M testosterone. It should be mentioned that the ER content varies with the growth conditions and that ER level was low in MCF-7 cells grown with 10% NCS + testosterone as can be seen in Fig. 2. PR-A and PR-B were expressed at high levels in MCF-7 cells grown with 10% NCS + testosterone and PR continued to be undetectable in the AI-resistant cell lines grown in medium with their respective AI, whereas Bcl-2 level varied between the AI-resistant cell lines (Fig. 3A). EGFR level was significantly higher in letrozole-resistant cell lines compared with MCF-7 and exemestane-resistant cell lines. HER2 level appeared to be slightly higher in resistant cells, whereas HER3 was expressed at level comparable with MCF-7. As also found in the initial analysis (Fig. 2), HER4 expression was higher in exemestane-resistant cell lines compared with MCF-7 and letrozole-resistant cell lines (Fig. 3A). In order to explore whether ER was functional, AI was withdrawn from the AI-resistant cell lines for one week and the cells were treated with estradiol, testosterone or testosterone in combination with AI for five days. Estradiol and testosterone induced expression of PR-A and PR-B as well as Bcl-2 in MCF-7 cells and the two AI-resistant cell lines, Let^R^-1 and Exe^R^-1 (Fig. 3B). The ER level was reduced in MCF-7, Let^R^-1 and Exe^R^-1 grown with estradiol and with testosterone as expected due to reduced stability of estradiol-bound ER. PR-A and PR-B were hardly detectable in MCF-7 cells grown with 10% NCS and when treated with exemestane and letrozole. PR-A and PR-B were expressed at low level in AI-resistant cells grown with 10% NCS and also expressed at low level in 10% NCS + testosterone and the respective AI. Bcl-2 was expressed at comparable level in control cells with 10% NCS and in cells grown with testosterone and AI (Fig. 3B). These data demonstrate that ER is functional in the AI-resistant cell lines and that the AIs inhibit the conversion of testosterone to estradiol.

Aromatase expression is highly regulated at the transcriptional level (12), and to investigate whether the AI-resistant cell lines displayed aberrant aromatase expression, we measured *CYP19A1* mRNA expression using real-time RT-PCR. The analysis revealed similar *CYP19A1* mRNA level in MCF-7, Let^R^-1, Let^R^-3, Exe^R^-1 and Exe^R^-3 cells (Fig. 3C).

### AI-resistant cell lines display low degree of androgen responsiveness and cross-resistance between letrozole and exemestane

**Figure 4:**
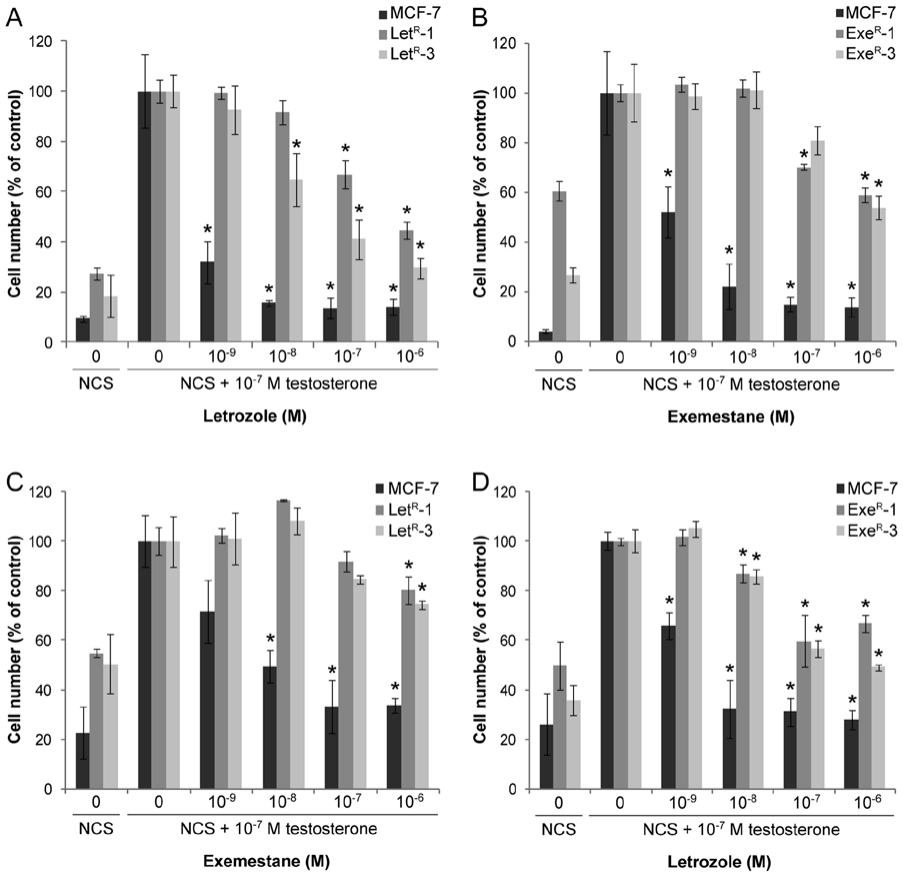
Effect of testosterone, testosterone plus letrozole or exemestane on growth of MCF-7 and AI-resistant cell lines. MCF-7 cells and the AI-resistant cell lines Let^R^-1, Let^R^-3, Exe^R^-1 and Exe^R^-3 withdrawn from testosterone and exemestane treatment for one week were grown for five days with 10% NCS, 10% NCS + 10^−7^ M testosterone and 10% NCS + 10^−7^ M testosterone in combination with the indicated concentrations of letrozole (A and D) or exemestane (B and C). Cell number was estimated by a colorimetric assay and expressed relative to the cell number in the cultures with 10% NCS + 10^−7^ M testosterone (control). Representative experiments of at least four experiments with four sample replicates are shown. Mean and SD are shown and the asterisks indicate statistically significant difference from the respective control cultures.

MCF-7 cells grown with 10% NCS displayed ~6–10-fold stimulation with 10^−7^ M testosterone, and letrozole and exemestane exerted a dose-dependent growth inhibition with maximum inhibition to the basal level with 10^−7^ M and 10^−6^ M AI (Fig. 4). The growth rate of the AI-resistant cell lines increased gradually during the first 15 weeks in medium with AI and then a constant cell population doubling time of ~30 h was achieved (corresponding to a weekly split ratio ~1:25). In comparison, the weekly split ratio for MCF-7 cells grown with 1% FCS is 1:40 (data not shown). To test whether the AI-resistant cell lines maintain the ability to be androgen-stimulated, testosterone and AI were withdrawn for one week from the AI-resistant cell lines before onset of the experiments shown in Fig. 4. Testosterone increased growth of all AI-resistant cell lines, mean stimulation was 2.7-, 2.5-, 1.4- and 3.3-fold for Let^R^-1, Let^R^-3, Exe^R^-1 and Exe^R^-3, respectively. A dose-dependent inhibition of the testosterone-stimulated cell growth was seen for AI-resistant cell lines treated with letrozole (Fig. 4A and D) and exemestane (Fig. 4B and C). AI treatment of the AI-resistant cell lines abrogated the testosterone-induced cell growth, but did not exert growth arrest as in MCF-7 cells. Noteworthy, the cell number in AI-resistant cell lines grown with 10% NCS was significantly higher than for MCF-7 cells and in contrast to MCF-7 cells, the AI-resistant cell lines could be propagated continuously in medium with 10% NCS (data not shown).

### Fulvestrant, but not tamoxifen, exerts complete growth arrest of AI-resistant cell lines

**Figure 5:**
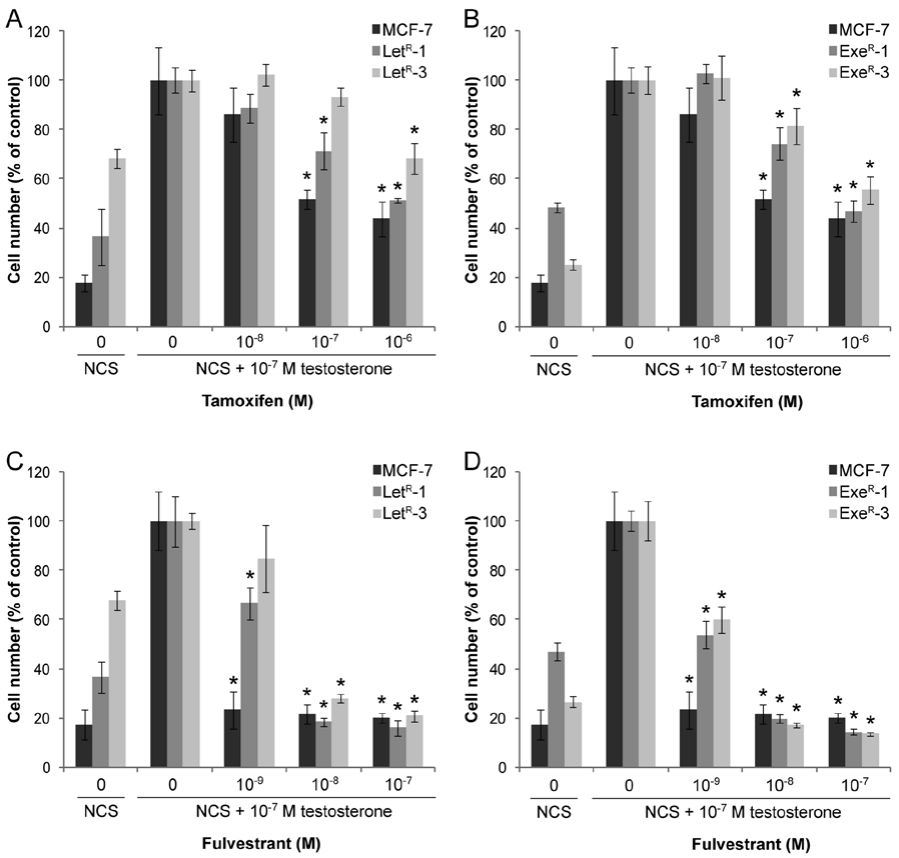
Effect of tamoxifen and fulvestrant on growth of MCF-7 and AI-resistant cell lines withdrawn from AI treatment. MCF-7 cells and the AI-resistant cell lines Let^R^-1, Let^R^-3, Exe^R^-1 and Exe^R^-3 withdrawn from testosterone and AI treatment for one week were grown for five days with 10% NCS, 10% NCS + 10^−7^ M testosterone and 10% NCS + 10^−7^ M testosterone in combination with the indicated concentrations of tamoxifen (A and B) or fulvestrant (C and D). Cell number was estimated by a colorimetric assay and expressed relative to the cell number in the cultures with 10% NCS + 10^−7^ M testosterone (control). Representative experiments of two independent experiments with four sample replicates are shown. Mean and SD are shown and the asterisks indicate statistically significant difference from the respective control cultures.

**Figure 6:**
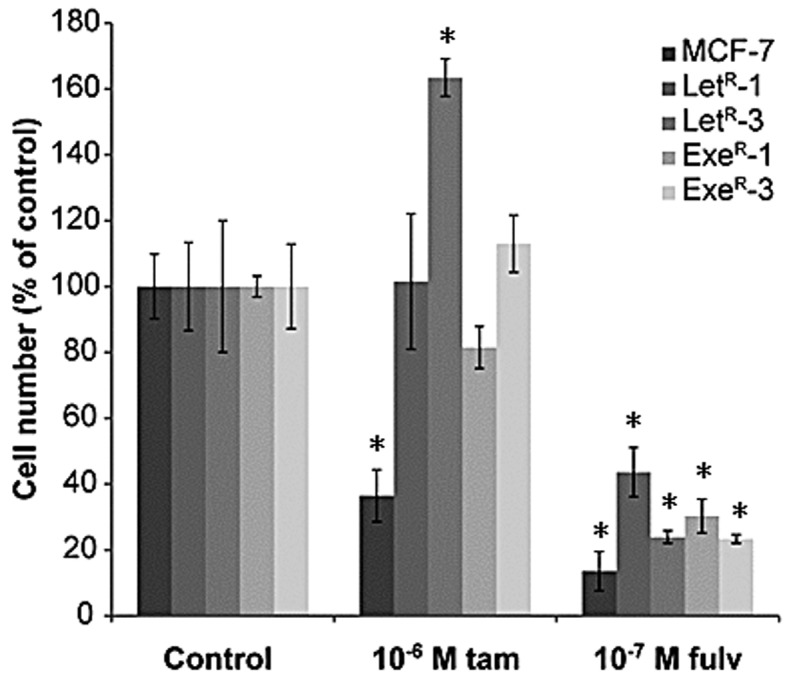
Effect of tamoxifen and fulvestrant on growth of MCF-7 and on AI-resistant cell lines in combination with AI. MCF-7 cells were grown in 10% NCS + 10^−7^ M testosterone alone or in combination with 10^−6^ M tamoxifen or 10^−7^ M fulvestrant. Let^R^-1, Let^R^-3, Exe^R^-1 and Exe^R^-3 were grown in their standard growth medium with the respective AI and in standard growth medium with the respective AI and with 10^−6^ M tamoxifen or 10^−7^ M fulvestrant for five days. Cell number was estimated by a colorimetric assay and expressed relative to the cell number in the control cultures (MCF-7 grown with 10% NCS + 10^−7^ M testosterone, Let^R^-1 and Let^R^-3 grown with 10% NCS + 10^−7^ M testosterone + 10^−6^ M letrozole, Exe^R^-1 and Exe^R^-3 grown with 10% NCS + 10^−7^ M testosterone + 10^−7^ M exemestane). Representative experiments of two independent experiments with four sample replicates are shown. Mean and SD are shown and the asterisks indicate statistically significant difference from the respective control cultures.

Tamoxifen is a weak estrogen antagonist and in medium with 10% NCS + 10^−7^ M testosterone, a dose-dependent growth inhibition of MCF-7 cells was seen. However, growth was not completely arrested after five days treatment with 10^−6^ M tamoxifen (Fig. 5A and B) as it is normally observed with MCF-7 cells grown in their standard medium with 1% FCS (41). Fulvestrant exerted complete growth arrest of MCF-7 cells at concentrations from 10^−9^ M (Fig. 5C and D). Dose-response growth experiments with antiestrogens were performed with AI-resistant cell lines withdrawn from AI treatment for one week (Fig. 5) and with cells grown with AI (Fig. 6). In AI-resistant cell lines grown with 10% NCS + 10^−7^ M testosterone and withdrawn from AI treatment for one week, tamoxifen induced a dose-dependent growth inhibition down to the level seen in 10% NCS (Fig. 5A and B). Fulvestrant also inhibited growth of AI-resistant cell lines in a dose-dependent manner, but in contrast to tamoxifen, fulvestrant reduced growth of the AI-resistant cell lines to below the level in NCS medium and down to the level seen in MCF-7 cells treated with 10^−7^ M fulvestrant (Fig. 5C and D). Tamoxifen treatment of AI-resistant cell lines grown in their standard growth medium with their respective AI had no effect or a stimulatory effect, whereas fulvestrant suppressed growth totally, as seen in MCF-7 cells (Fig. 6). To determine whether AI-resistant cell lines were completely growth arrested with fulvestrant, cultures with Let^R^-1 and Exe^R^-1 cells were treated with 10^−7^ M fulvestrant for one week, split and treated for further one week with fulvestrant. After a total of 14 days of treatment with 10^−7^ M fulvestrant, no viable Let^R^-1 or Exe^R^-1 cells could be detected.

## Discussion

Adjuvant treatment with AIs postpones or abrogates development of advanced disease, and patients with advanced disease benefit from treatment. However, most patients with advanced disease will eventually progress, and some patients receiving adjuvant AIs progress during therapy. To find new treatment options and new biomarkers for resistant tumors, it is important to gain knowledge of the molecular mechanisms involved in resistance to AIs. In this report, we describe a new series of breast cancer cell lines with acquired resistance to the clinically relevant AIs letrozole, exemestane and anastrozole, which may be useful models for studies of the resistance mechanisms. The resistant cell lines were developed from MCF-7 cells grown under conditions at which the extracellular estrogen supply via serum was low and growth stimulation was mediated via conversion of testosterone to estradiol by the endogenous aromatase enzyme. Long-term treatment of MCF-7 cells with letrozole, exemestane or anastrozole appeared to inhibit growth completely, but some cells survived treatment and gave rise to outgrowth of colonies, which could be isolated and propagated continuously in presence of the respective AI. Initially, the growth rate was slow, but a gradual increase in growth rate was observed during the first 15 weeks of treatment until constant growth rate was achieved. We believe that the surviving colonies arise from cells with inherent resistance, whereas acquired changes may be responsible for the observed increase in growth rate during long-term propagation.

A switch from ER-driven growth to involvement of HER receptor-driven growth has been described for antiestrogen-resistant breast cancer cells (41–44), and model systems have indicated that resistance to AIs may involve cross-talk between ER and growth factor signaling pathways, and ligand-independent activation of ER (27, 45–47). The initial analysis of expression of ER, estrogen-regulated proteins and HER receptors revealed that ER expression was severely reduced in only one (8%) of twelve AI-resistant cell lines, and a similar low fraction of patients were found to be ER-negative at relapse or progression after AI treatment (11). In general, HER receptor level was higher in AI-resistant cell lines than in parental cells, in particular EGFR was increased in the letrozole-resistant cell lines and HER4 was increased in exemestane-resistant cell lines, pointing to involvement of the HER receptor system in AI resistance. Two letrozole and two exemestane resistant cell lines were selected for further studies and the expression pattern of ER, ER-regulated proteins and HER receptors did not change significantly during longer time propagation of the cell lines. ER was functional with respect to stimulation of expression of PR and Bcl-2, and ER protein expression was reduced in both MCF-7 cells and AI-resistant cell lines grown with estradiol and testosterone. Downregulation of ER expression in MCF-7 cells grown with estradiol and with testosterone has been observed before (35,48) and may be explained by destabilization of the ER protein upon binding to estradiol (49). The testosterone-induced expression of the estrogen-regulated proteins PR-A, PR-B and Bcl-2 in the AI-resistant cell lines verified that AI-resistant cells were able to convert testosterone to estradiol via the aromatase enzyme. AI treatment significantly reduced PR expression in AI-resistant cell lines, but PR expression was not totally blocked as in MCF-7 cells, suggesting that the AIs may not completely block the aromatase activity or that PR may be induced by ligand-independent activation of ER. A similar low PR expression was found in AI-resistant cell lines grown with NCS alone, supporting a ligand-independent activation of ER. Upregulation of aroma-tase gene expression could result in insufficient inhibition of the aromatase enzyme in the AI-resistant cells and explain the transcriptional activity of ER. However, the observed similar level of *CYP19A1* mRNA in parental MCF-7 cells and in the four tested AI-resistant cell lines does not support this mechanism of resistance.

Cell number in both the letrozole- and the exemestane-resistant cell lines increased 20–25 times during one week in the standard growth medium with the respective AI, and all resistant cell lines displayed a low degree of responsiveness to growth stimulation with testosterone when AI and testosterone were withdrawn for one week. The ability of the AI-resistant cell lines to grow continuously in presence of AI and also in medium with 10% NCS demonstrates that the cell lines have acquired ability to grow without estrogen stimulation, whereas the observed growth response to testosterone indicates that AI-resistant cell lines have progressed from estrogen-dependent to estrogen-responsive cell growth. Letrozole, exemestane and tamoxifen could abrogate the testosterone-mediated growth stimulation, but could not reduce growth to below the level in standard growth medium with AI. In contrast, fulvestrant inhibited growth of the AI-resistant cell lines to below the level in 10% NCS and down to the level of MCF-7 cells which were completely growth arrested by treatment with fulvestrant. To investigate whether fulvestrant completely blocked growth of AI-resistant cell lines, AI-resistant cells treated for one week with fulvestrant were trypsinized and seeded in new culture flasks, but no growth was observed, demonstrating that fulvestrant exerted complete growth arrest of AI-resistant cell lines and supporting that growth of the AI-resistant cell lines depends primarily on ER. The severe growth inhibition seen with fulvestrant treatment of AI-resistant cell lines in their standard growth medium with AI also supports that ER drives growth of the AI-resistant cell lines. The importance of ER-mediated growth and effect of fulvestrant treatment have also been found in exemestane-resistant cell lines derived from aromatase overexpressing MCF-7 cells (34), in MCF-7 breast tumor xenograft with letrozole-resistant aromatase overexpressing cells (50), and in the LTED model system (26). Clinical benefit rates of 32% were found in two phase III studies with fulvestrant treatment of patients with advanced disease after progression on a non-steroidal AI (51,52), supporting that ER may also be an important driver of metastatic breast cancer cells in patients relapsing from AI therapy.

Letrozole and exemestane abrogated the testosterone-mediated growth of both letrozole- and exemestane-resistant cell lines but could not arrest cell growth, demonstrating cross-resistance between the non-steroidal AI letrozole and the steroidal AI exemestane. This is in contrast to clinical studies in which sequential treatment from a non-steroidal to a steroidal AI or *vice versa* has resulted in clinical benefit for 30–50% of the patients (53). It should be mentioned that the studies included low number of patients (mean 54) and objective response rates were low, average in 8 studies was 11%. Furthermore, mechanisms explaining lack of cross-resistance have not been documented (53), but lack of effective uptake in tumor tissue of particular compounds has been suggested as a possibility (54) and patients may metabolize the compounds differently. Such mechanisms will not be disclosed in our *in vitro* cell culture model.

Tamoxifen was able to abrogate the testosterone-mediated growth stimulation of the AI-resistant cell lines but could not reduce growth further, demonstrating that tamoxifen is able to inhibit the estrogen-mediated but not the presumed ligand-independent activation of ER. In line with this, we have recently found that ER is the main driver of growth of tamoxifen-resistant cell lines (41). The multi-targeting kinase inhibitors sorafenib and nilotinib could restore the sensitivity of tamoxifen-resistant cell lines to tamoxifen, indicating that growth of tamoxifen-resistant cells occurs via ligand-independent activation of the ER (55). More direct evidence of the inability of tamoxifen to inhibit ligand-independently activated ER was obtained in a study showing that Aurora kinase A upon phosphorylation of ER renders breast cancer cells less sensitive to treatment with tamoxifen (56). In agreement with this, we have shown that Aurora kinase A plays a major role for growth of tamoxifen-resistant breast cancer cell lines, and that inhibition of Aurora kinase A restores the sensitivity to tamoxifen treatment (57). Whether Aurora kinase A plays a major role by ligand-independent activation of ER in AI resistance is under investigation. It should be mentioned that about one third of patients recurring from tamoxifen treatment benefit from treatment with AI (58,59), showing that different mechanisms for tamoxifen and AI resistance also exist.

In conclusion, this report presents a large series of AI-resistant breast cancer cell lines derived from MCF-7 cells, which can be used as models to unravel the molecular mechanisms for growth of breast cancer in patients who recur after an initial response to AI therapy. We found that the majority of AI-resistant cell lines maintained ER expression and function, whereas HER receptor expression was increased. The complete growth inhibition of the AI-resistant cell lines by treatment with the ER down modulator fulvestrant demonstrates that ER is the main driver of growth of AI-resistant cell lines, supporting the potential of fulvestrant therapy for AI-resistant breast cancer.
